# Determinants and spatial factors of anemia in women of reproductive age in Democratic Republic of Congo (drc): a Bayesian multilevel ordinal logistic regression model approach

**DOI:** 10.1186/s12889-023-17554-y

**Published:** 2024-01-17

**Authors:** Martin Abysina Soda, Eugénie Kabali Hamuli, Salomon Agasa Batina, Ngianga-Bakwin Kandala

**Affiliations:** 1 Section de Sciences Infirmières Institut Supérieur des Techniques Médicales de Kisangani, Kisangani, Democratic Republic of the Congo; 2 Institut Supérieur Des Techniques Médicales de Kinshasa, Kinshasa, Democratic Republic of the Congo; 3 Département de Médecine Interne, Université de Kisangani, Kisangani, Democratic Republic of the Congo; 4 Department of Epidemiology and Biostatistics, Western University, London, ON Canada; 5 Division of Epidemiology and Biostatistics, School of Public Health, University of Witwatersrand, Witwatersrand, South Africa

**Keywords:** Determinants and spatial factors, Anemia in women of reproductive age, Multilevel and spatial Bayesian ordinal logistic regression model

## Abstract

**Background:**

As a global public health problem, anemia affects more than 400 million women of reproductive age worldwide, mostly in Africa and India. In the DRC, the prevalence of anemia has decreased slightly from 52.9% in 2007, to 46.4% in 2012 and 42.4% in 2019. However, there is considerable regional variation in its distribution. The aim of this study is to determine the factors contributing to anemia in women of reproductive age and to explore its spatial distribution in the DRC.

**Methods:**

Based on the Bayesian Multilevel Spatial Ordinal Logistic Regression Model, we used the 2013 Democratic Republic of Congo Demographic and Health Survey (DHS-DRC II) data to investigate individual and environmental characteristics contributing to the development of anemia in women of reproductive age and the mapping of anemia in terms of residual spatial effects.

**Results:**

Age, pregnancy status, body mass index, education level, current breastfeeding, current marital status, contraceptive and insecticide-treated net use, source of drinking water supply and toilet/latrine use including the province of residence were the factors contributing to anemia in women of reproductive age in DRC. With Global Moran's I = -0.00279, *p*-value ≥ 0.05, the spatial distribution of anemia in women of reproductive age in DRC results from random spatial processes. Thus, the observed spatial pattern is completely random.

**Conclusion:**

The Bayesian Multilevel Spatial Ordinal Logistic Regression statistical model is able to adjust for risk and spatial factors of anemia in women of reproductive age in DRC highlighting the combined role of individual and environmental factors in the development of anemia in DRC.

## Introduction

As a global public health problem, anemia impairs women's health and well-being and increases the risk of adverse maternal and newborn outcomes in low- and middle-income countries affecting half a billion women of reproductive age worldwide. In 2011, 29% of non-pregnant women and 38% of pregnant women were anemic [1]. About 20% of maternal deaths are caused by anemia [2]. Pregnancy, intestinal infestation, low level of education, use of an unimproved water source, low wealth index and underweight are listed as risk factors [3].

Anemia in women of reproductive age has consequences such as premature delivery [4], miscarriage [5], low birth weight [6], stunting of the child's growth [7], Impaired cognitive function [8] and increased susceptibility to infection [4].

Africa and India have the highest rates of anemia, with nearly 50% of women affected, including 40% of maternal deaths [9]. In DRC, the prevalence of anemia in women has decreased slightly from 52.9% in 2007 to 46.4% in 2012 and 42.4% in 2019 [10].

To reduce the burden of anemia, it is necessary to generate adequate evidence in terms of the role and contribution of individual and household factors as well as the geographic risk profile of anemia [3]. Ordinal data such as altitude-adjusted hemoglobin levels are used when measurements are limited to categories [11]. However, the Multilevel Model describes observations with a nested nature: women of reproductive age are nested within households and communities including the environment [12].

Based on Bayes' theorem, Bayesian statistics analyzes data and estimating the observed and unobserved parameters of a statistical model with a joint probability distribution, known as the "prior distribution" and the "data distribution" [13]. A typical Bayesian process involves capturing available knowledge about the statistical model parameter via the prior distribution, usually before data collection, determining the likelihood function using information about the available parameters of the observed data, and combining both the prior distribution and the likelihood function [14]. Bayesian analysis answers research questions by expressing uncertainty about unknown parameters using probabilities. It is based on the fundamental assumption that not only the outcome of interest, but also all unknown parameters of a statistical model are essentially random and subject to prior beliefs [15]. Using Bayes' theorem in the form of an a posteriori distribution, inferences are made to reflect updated knowledge and balance prior knowledge with observed data. Bayesian inferences are optimal when averaged over the joint probability distribution and inference for these quantities is based on the conditional distribution given the observed data [16].

Geographic differences in the causes of anemia are partially explained by the large-scale variability of environmental factors, particularly nutritional and infectious causes. Environmental factors in anemia tend to show a high degree of spatial dependence, that is, geographic clustering [17]. The study of geographic heterogeneity in a health outcome benefits from the multilevel or spatial mixed model. In the multilevel model, geographic heterogeneity is modeled as a random effect [18]. Whereas in the spatial mixed model, geographic heterogeneity is assessed by specifying a spatial correlation structure for the individual residuals. A comparative study of a multilevel model and a spatial mixed model to investigate the effects of location on health outcomes showed lower deviance for the spatial mixed model than for the multilevel model, and that Moran's I statistic showed residual spatial autocorrelation not accounted for by the multilevel model [19]. Ignoring heterogeneity in statistical studies can lead to biased parameter estimates [20].

Many studies have explored the factors affecting anemia in women of reproductive age using DHS-DRC I and II data. Given the diversity of the Congolese population in terms of culture, ethnicity, and geographic location, distinct characteristics such as dietary habits, lifestyle, and socioeconomic status related to geographic regions are unique and pose the risk of geographic heterogeneity in the causes of anemia in women of reproductive age. Studies of geographic heterogeneity in modeling anemia with the flexible ordinal approach are also very few worldwide and nonexistent in DRC.

Our study aims to specifically answer two questions:

- What is the likelihood that a woman of reproductive age living in the DRC will develop anemia?
- What is the likelihood that one province in the DRC is more likely to have significantly more cases of anemia in women of reproductive age than another?

The main objective of this study is to determine the factors contributing to anemia in women of reproductive age and to explore the spatial distribution of this condition.

The contribution of this study is the application of the multilevel and spatial Bayesian ordinal logistic regression model. The study has the advantage of identifying and mapping anemia in women of reproductive age in DRC in terms of residual spatial effects. This study will have important implications for targeting policy as well as for finding omitted variables that could explain residual spatial patterns. Exploring patterns of factors affecting anemia by geographic region is therefore essential to inform policy for targeted anemia control and prevention programs.

## Methods

### Study area

Our study focused on the DRC, Africa's second largest country after Algeria. The DRC stretches from the Atlantic Ocean to the eastern plateau, covering most of the Congo River basin. The country shares borders with nine neighboring countries, including the enclave of Cabinda. Located in Central Africa, the DRC is crossed by the equator and stretches across the Congo Basin.

### Study data source and sampling strategy

Our study is drawn from the 2013–2014 Demographic and Health Survey (DHS-DRC II) data, which applied a three-stage stratified cluster sampling technique. 540 clusters were drawn as Primary Sampling Units representing neighborhoods in urban areas or villages in rural areas. While 18,360 households, including 5,474 in urban areas in 161 clusters and 12,886 in rural areas in 379 clusters, were drawn as Secondary Sample Units. Thus, all women aged 15–49 usually living in the selected households, or present on the night before the survey, were eligible for the survey. In addition, in a subsample of every other household, a hemoglobin test was administered to estimate the prevalence of anemia among all women identified in the households [10].

The strategy of collection by cascades was envisaged by survey pools whose number of clusters varied from 10 in the Mweka pool to 20 in the Lubumbashi pool, with the exception of 36 clusters carried out in the pool in the city of Kinshasa Province. Thus, 34 pools were identified [10].

Data collection was done through a questionnaire whose content was developed and translated into the four main national languages: Kikongo, Lingala, Swahili and Tshiluba. In the 18,171 households surveyed, 19,097 women aged 15–49 were eligible for the individual survey, and 18,827 of them were successfully interviewed, representing a response rate of 99%, slightly higher in rural areas than in urban areas (99% versus 98%) [10]. Our analysis was based on a sub-sample of women (*N* = 9280) that were tested for anemia.

### Study variables

#### Outcome variable

Blood samples for the anemia test collected from women aged 15–49 years who voluntarily consented to be tested were taken from a drop of blood taken by finger prick and collected in a microcuvette. Hemoglobin analysis was performed on site using a battery-operated portable HemoCue analyzer. The complete sampling procedure and anemia test data are available in the full DHS-DRC II report [10].

According to WHO, for pregnant and non-pregnant women aged 15–49 years, any form of anemia was defined as a hemoglobin concentration < 120 g/L and < 110 g/L, respectively. The four levels of altitude-adjusted hemoglobin based on the WHO hemoglobin thresholds for diagnosis of anemia were categorized into i) No anemia, i.e., 120 g/L and above, ii) Mild anemia, 110 to 119 g/L), iii) Moderate anemia, 80 to 109 g/L, and iv) Severe anemia, < 80 g/L [21].

#### Predictive variables

Predictive variables for anemia were selected based on the literature review. In 2017, WHO described the determinants of anemia, including biological, infection and inflammation-related determinants, genetic disorders of hemoglobin, and social, behavioral, and environmental determinants [22]. Predictors of anemia include factors at the individual, household and community levels.

At the individual level, eight factors were identified. These were age, pregnancy status, nutritional status, breastfeeding status, level of education, occupation and use of contraceptives and insecticide-treated nets. Household-level factors were household economic well-being index, access to drinking water and household toilets. Community factors were place of residence and province.

### Data analysis

#### Data management and processing

Data processing and analysis was performed using Stata/BE 17.0 (StataCorp). In order to obtain reliable statistical estimates, complex survey data were weighted using sample weight, primary sampling unit, and strata prior to any statistical analysis. Survey weights were used in both univariate and bivariate analysis to ensure representativeness and to account for nonresponse. Due to practical difficulty, weights were not used in the multilevel Bayesian modeling [23].

Weighted frequencies, weighted percentage, mean, standard deviation, and standard deviation of the mean, or standard error, were used for descriptive analysis. Pearson's χ2 test for categorical variables was used. Bayesian Multilevel Ordinal Logistic Regression analysis was performed to estimate the posterior Odds ratio and associated 95% credible intervals.

#### Common techniques for handling missing data

We used leastwise deletion of observations, a Missing Completely At Random (MCAR) mechanism, which is one of three types developed to handle missing values. Thus, we removed all incomplete observations from the analysis. The disadvantage of this method is the reduction of the sample size [24]. Thus, of the 9,461 initial observations, 9,280 remained which was the subject of our study that is 98.08%.

#### Bayesian multilevel ordinal logistic regression

Referring to the DHS data set based on multi-stage stratified cluster sampling, the structure of the data in the population was hierarchical. The grouping of data points into geographical regions provides a natural two-level hierarchical structure of the data, i.e. women are nested within provinces [25]. In this analysis, we proposed the Multilevel mixed-effects ordered logistic regression Model to fit the woman's two levels of anemia by one or more independent variables, with random intercepts by id, using default normal priors, flat priors for the cut points, and default inverse gamma priors for the variance of the random intercepts. Next, we displayed the posterior odds ratio and associated 95% credible intervals [26]. This Model contains both fixed effects and random effects at different levels and analyzes the effects of the woman's individual characteristics, such as age and body mass index, and the effects of the characteristics of the environment experienced by the woman, such as the household's economic well-being index and the woman's household's access to drinking water [27].

We used the "Bayesian equal tail CrI" method that returns threshold values of the posterior distribution to represent a credibility interval with the 95% probability of interest of the mass of the distribution around the center of the distribution [28].

Considering Two-Level Model, where for a series of M independent clusters, and subject to a set of fixed effects xij, a set of cutpoints k, and a set of random effects uj, the cumulative probability that the response was in a category greater than k was [29]:

1for j = 1,…, M clusters, with cluster j consisting of i = 1,…, nj observations. The cutpoints k are labeled k_1_, k_2_,…, k_k-1_, where k is the number of possible outcomes. H(.) is the logistic cumulative distribution function that represents cumulative probability.

The 1 × p row vector x_ij_ are the covariates for the fixed effects. x_ij_ does not contain a constant term because its effect is absorbed into the cutpoints. For notational convenience, we suppress the dependence of y_ij_ on x_ij_.

The 1 × q vector z_ij_ are the covariates corresponding to the random effects and can be used to represent both random intercepts and random coefficients. The random effects u_j_ are M realizations from a multivariate normal distribution with mean 0 and q x q variance matrix. They are not directly estimated as model parameters but are instead summarized according to the unique elements of Σ, known as variance components.

From [1], we can derive the probability of observing outcome k as follows:

where k_0_ was taken as -∞ and k_K_ was taken as + ∞.

From this, the model can also be written in terms of latent linear response, where the observed ordinal responses yij are generated from the latent continuous responses, such that:





And

$${{\text{y}}}_{{\text{ij}}}=\left\{\begin{array}{c}\begin{array}{c}\mathrm{Non anemic if hemoglobin level }> 11.9\mathrm{ g}/{\text{dl}}\\ \mathrm{Mild anemic if hemoglobin level }10.0\mathrm{ to }11.9\mathrm{ g}/{\text{dl}}\end{array}\\ \begin{array}{c}\mathrm{Moderate anemic if hemoglobin level }7.0\mathrm{ to }9.9\mathrm{ g}/{\text{dl}}\\ \mathrm{Severe anemic if hemoglobin level }< 7.0\mathrm{ g}/{\text{dl}}\end{array}\end{array}\right.$$

The errors ϵ_it_ are distributed as logistic with mean 0 and variance π^2^/3 and are independent of u_j_.

We also assumed informative precedence, i.e. Normal(0,10); μprovince is the province-level effect, which follows a normal distribution with a mean of zero. The model was fitted with Stata 17.0 BE-Basic Edition software for the "Bayesian multilevel ordered logistic regression" command using "Random-walk Metropolis–Hastings sampling" for MCMC iterations = 12,500 and Burn-in = 2,500.

#### Analysis of spatial autocorrelation

Spatial autocorrelation analysis determines the systematic spatial variation in a mapped variable. When adjacent observations have similar data values, the map shows positive spatial autocorrelation. However, when these adjacent observations tend to have highly contrasting values, the map shows negative spatial autocorrelation instead [30]. Several statistical techniques exist to detect the presence of these values. Currently, spatial autocorrelation is tested using the global Moran index (Moran's I). The value of Moran's I is between -1 and 1. A value close to 1 indicates strong positive spatial autocorrelation (clustered anemia), while a value close to -1 indicates strong negative spatial autocorrelation (scattered anemia). If Moran's I is close to 0, it indicates that there is no spatial autocorrelation. A statistically significant Moran's I (p < 0.05) leads to the rejection of the null hypothesis, as the anemia was randomly distributed, and shows the presence of spatial autocorrelation [31]. Hotspot analysis was performed using the Gettis-OrdGi* statistic.

## Results

The exploratory results for the sampled population presented in Table 1 estimate that 38.45% of DRC women of reproductive age in the sample are anemic (weighted sample).

**Table 1:** Description and categorization of the dependent variable

Anemia	Weighted number	Weighted percentage
Severe anemia	38	0.34
Moderate anemia	884	8.53
Mild anemia	2872	29.58
No anemia	5486	61.55

The results in Table 2 indicate that pregnancy status, body mass index, education level, age at first birth, current breastfeeding and contraceptive use are potential factors associated with anemia, although these results do not control for the impact of other factors.

**Table 2:** Bivariate analysis of factors associated with anemia in women of reproductive age (weighted)

Predictors	Anemia			No anemia	Total
	**Severe** Nw(w%)	**Moderate** Nw(w%)	**Mild** Nw(w%)	Nw(w%)	Nw(w%)
**Age (years)**	**Chi2(9) = 6.0977**				***P* = 0.7295**
< 20	3 (8.41)	50 (6.74)	178 (6.23)	316 (5.17)	547 (5.63)
20–29	20 (4.895)	448 (54.21)	1,450 (49.26)	2,787 (50.83)	4,705 (50.65)
30–39	9 (26.91)	315 (31.50)	988 (36.25)	1,869 (34.85)	3,181 (34.95)
40–49	6 (15.73)	71 (7.55)	256 (8.26)	514 (9.15)	847 (8.77)
**Pregnancy**	**Chi2(3) = 47.0253**				***P* < 0.001**
No Yes	24 (57.61) 14 (42.39)	578 (64.57) 306 (35.43)	2,572 (89.72) 300 (10.28)	4,729 (86.66) 757 (13.34)	7,903 (85.58) 1,377 (14.42)
**Body mass index**	**Chi2(9) = 25.1563**				***P* = 0.0033**
Normal weight Underweight Overweight Obese	29 (82.37) 8 (14.18) 1 (3.45) 0 (0.00)	677 (75.14) 106 (10.95) 87 (12.33) 14 (1.59)	2,161 (72.88) 382 (14.38) 261 (9.87) 68 (2.87)	3,954 (71.34) 690 (11.17) 712 (14.35) 130 (3.15)	6,821 (72.16) 1,186 (12.11) 1,061 (12.81) 212 (2.92)
**Level of education**	**Chi2(9) = 21.6940**				***P* = 0.0111**
No education	6 (13.97)	173 (15.26)	583 (17.10)	1,235 (21.30)	1,997 (19.52)
Primary	17 (43.65)	428 (46.18)	1,298 (43.72)	2,460 (44.13)	4,203 (44.18)
Secondary	15 (42.38)	274 (36.79)	977 (38.54)	1,745 (33.68)	3,011 (35.41)
Higher	0 (0.00)	9 (1.77)	14 (0.64)	46 (0.90)	69 (0.89)
**Currently breastfeeding**	**Chi2(3) = 17.7695**				***P* < 0.001**
Non-breastfeeding women	15 (49.00)	390 (43.34)	888 (30.47)	1,698 (30.11)	2,991 (31.41)
Breastfeeding women	23 (51.00)	494 (56.66)	1984 (69.53)	3,788 (69.89)	6,289 (68.59)
**Current marital status**	**Chi2(6) = 11.2088**				***P* = 0.0846**
Never in a union	2 (4.08)	29 (2.54)	138 (5.38)	288 (3.99)	397 (4.28)
Married/Living with a partner	30 (80.44)	802 (91.80)	2,475 (85.39)	4,776 (87.50)	8,083 (87.22)
Widowed/ Divorced/Separated	6 (15.48)	53 (5.67)	259 (9.23)	482 (8.50)	800 (8.50)
**Recent work**	**Chi2(3) = 0.8029**				***P* = 0.8487**
No recent work	6 (15.87)	218 (25.38)	662 (24.12)	1,308 (24.74)	2,194 (24.58)
Working	32 (84.13)	666 (74.62)	2,210 (75.88)	4,178 (75.26)	7,086 (75.42)
**Contraceptive use**	**Chi2(3) = 10.5295**				***P* = 0.0153**
Not a user	37 (95.15)	774 (85.44)	2,369 (79.85)	4,465 (79.54)	7,645 (80.19)
User	1 (4.85)	110 (14.56)	503 (20.15)	1,021 (20.46)	1,635 (19.81)
ITN* user	**Chi2(3) = 5.3733**				***P* = 0.1480**
Does not sleep on ITN	19 (44.23)	331 (38.40)	1,113 (37.50)	1,931 (33.23)	3,394 (34.97)
Sleeps on ITN	19 (55.77)	553 (61.60)	1,759 (62.50)	3,555 (66.77)	5,886 (65.03)
**Household Well-Being Index**	**Chi2(6) = 9.5767**				***P* = 0.1464**
Poor Household Average household Rich household	25 (52.81) 2 (4.68) 11 (42.51)	476 (46.87) 170 (17.41) 238 (35.72)	1,422 (45.64) 583 (20.52) 867 (33.84)	2,750 (45.82) 1,178 (21.45) 1,558 (32.73)	4,673 (45.88) 1,933 (20.77) 2,674 (33.35)
**Source of drinking water**	**Chi2(3) = 1.3976**				***P* = 0.7062**
Unimproved source Improved source	28 (62.67) 10 (37.33)	588 (56.73) 296 (43.27)	1,791 (56.44) 1,081 (43.56)	3,357 (53.66) 2,129 (46.34)	5,764 (54.77) 3,516 (45.23)
**Types of toilets/latrines**	**Chi2(3) = 1.3648**				***P* = 0.7139**
Unimproved toilets Improved toilets	27 (70.01) 11 (29.99)	592 (59.45) 292 (40.55)	1,822 (60.87) 1,050 (39.13)	3,550 (62.79) 1,956 (37.21)	5,971 (61.96) 3,309 (38.04)
**Natural environment**	**Chi2(3) = 3,7832**				***P* = 0.2872**
Urban environment Rural environment	12 (37.20) 26 (62.80)	247 (34.57) 637 (65.43)	837 (30.04) 2,035 (69.96)	1538 (28.09) 3948 (71.91)	2,634 (29.25) 6,646 (70.75)

*Nw* Weighted number, *%w* Weighted percentage, *ITN* insecticide-treated nets

ITN* insecticide-treated nets

Referring to Table 3, Multilevel Bayesian Ordinal Logistic Regression analysis indicates that age, pregnancy status, Body Mass Index, education level, current breastfeeding, current marital status, use of contraceptives and insecticide-treated nets, source of drinking water and use of toilets or latrines are significantly associated with anemia in women of reproductive age in the Democratic Republic of Congo.

**Table 3:** Multilevel Bayesian Ordinal Logistic Regression Model

Determinants	Posterior OR*	SD*	[95% credible interval]
**Age** (years) Ref*: < 20			
20–29	1.0863	0.0408	[1.0114; 1.1749]
30–39	1.0057	0.0466	[0.9145; 1.1004]
40–49	1.1055	0.0457	[1.0191; 1.1970]
**Pregnancy** Ref *: No			
Yes	0.7070	0.0301	[0.6406; 0.7650]
**Body mass index** Ref*: Normal weight			
Underweight	1.0474	0.0394	[0.9722; 1.1260]
Overweight	1.3926	0.0641	[1.2640; 1.5185]
Obese	1.1256	0.0615	[1.0029; 1.2487]
**Level of education** Ref*: No education			
Primary	0.9356	0.0351	[0.8708; 1.0093]
Secondary/Higher	0.9071	0.0372	[0.8366; 0.9824]
**Currently breastfeeding** Ref*: No-breastfeeding			
Breastfeeding women	1.0918	0.0305	[1.0332; 1.1525]
**Current marital status** Ref*: Never in a union			
Married/Living with a partner	1.0367	0.0381	[0.9677; 1.1128]
Widowed/ Divorced/Separated	1.0883	0.0641	[0.9623; 1.2293]
**Contraceptive use** Ref*: Not user			
User	0.7526	0.0317	[0.6991; 0.8209]
ITN* user Ref*: Doesn’t sleeps on ITN			
Sleeps on ITN	1.0715	0.0052	[1.0217; 1.1345]
**Household Well-Being Index** Ref*: Poor Household			
Average household	1.0704	0.0320	[1.0052; 1.1260]
Rich household	0.9672	0.0440	[0.8822; 1.0597]
**Source of drinking water** Ref*: Unimproved source			
Improved source	1.1512	0.0436	[1.0680; 1.2400]
Types of toilets/latrines Ref*: Unimproved toilets			
Improved toilets	0.9364	0.0298	[0.8769; 0.9946]
**Natural environment** Ref*: Urban environment			
Rural environment	1.0217	0.0434	[0.9416; 1.1065]
**Province**	0.1365	0.0447	[0.0719; 0.2443]

Posterior OR*: Odd ratio. SD* standard deviation, Ref* Reference, ITN* insecticide-treated nets

Anemia in women of childbearing age evolves in an inverted "U" shape with age. For a one-unit increase in age, women aged 20–29 have a 9% increased risk of developing anemia compared with younger women (OR = 1.09; CrI95%: 1.01–1.17), while women aged 40 and over have an 11% increased risk of developing anemia compared with younger women (OR = 1.11; CrI95%: 1.01–1.19), given that other variables are held constant in the model.

Pregnancy status determines anemia risk in women of childbearing age. Pregnant women have a 29% lower risk of developing anemia than non-pregnant women (OR = 0.70; CrI95%: 0.64–0.76), given that other variables are held constant in the model.

The risk of anemia increases with body mass index in women of childbearing age. For a one-unit increase in body mass index, overweight and obese women are respectively 39% and 13% more likely to develop anemia than those of normal weight (OR = 1.39; CrI95%: 1.26–1.51 and OR = 1.12; CrI95%: 1.002–1.24).

The risk of anemia in women of childbearing age decreases with increasing level of education. For a one-unit increase in education level, women with secondary/university education have a 9% lower risk of anemia than women with no education (OR = 0.90; CrI95%: 0.83–0.98).

Continuous breastfeeding determines the risk of anemia in women of childbearing age. Breastfeeding women have a 9% increased risk of developing anemia compared to non-breastfeeding women (OR = 1.09; CrI95%: 1.03–1.15).

The use of contraceptive methods determines a woman's risk of anemia during her reproductive years. Women using contraceptive methods to space births have a 25% lower risk of developing anemia than those not using them (OR = 0.75; CrI95%: 0.69–0.82).

Insecticide-treated net use determines the risk of anemia in women of childbearing age. Unexpectedly, women sleeping under insecticide-treated mosquito nets have a 7% increased risk of developing anemia compared to those not using them (OR = 1.07; CrI95%: 1.02–1.13).

Household wealth influences anemia in women. Women from middle-income households have a 7% higher risk of developing anemia than those from poor households (OR = 1.07; CrI95%: 1.005–1.12).

Sources of drinking water had significant impact on women's anemic status. Unexpectedly, women whose drinking water came from improved sources were 15% more likely to develop anemia than those whose water came from unimproved sources (OR = 1.15; CrI95%: 1.06–1.24).

Sanitation facilities have a negative impact on the anemic status of women of childbearing age. Women using improved latrines have a 6% lower risk of developing anemia than women using unimproved latrines (OR = 0.93; CrI95%: 0.87–0.99).

Referring to Table 4, the spatial distribution of anemia in women of reproductive age in the DRC was identified as dispersed (Global Moran's I = -0.00279, p-value ≥ 0.05). Not being able to reject the null hypothesis, it is entirely possible that the spatial distribution of attribute values of the features is the result of random spatial processes. Thus, the observed spatial pattern of values could well be one of countless possible scenarios of a completely random structure.

**Table 4:** Moran’s I statistics and Getis-Ord G*i(d) Statistics

Variable	Moran's I	E(I)	SE(I)	Z(I)	*p*-value
Anemia	-0,00279	-0,00532	0,01111	0,22,762	0,81,994
	**z < = -2.58**	**-2.58 < z < = -1.96**	**-1.96 < z < 1.96**	**1.96 < = z < 2.58**	**2.58 < = z**
	0	16	173	0	0

**Fig. 1:**
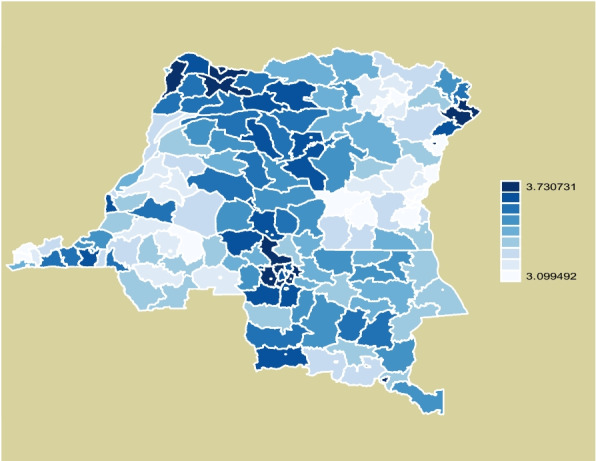
Clusters of anemia in women of reproductive age in DRC. Legend: From severe anemia (dark blue) to no anemia (white)

Based on Gettis-OrdGi statistical analysis, this study identified 173 hot spots, or clusters of anemia, and 16 cold spots, no clustering of anemia, of women of reproductive age in DRC. These identifications are shown in dark blue to white in Fig. 1 below.

## Discussion

The overall prevalence of anemia in this study is 38.45%, a very high level compared to the global prevalence of 29.9%. The prevalence in our study is variable from that reported by WHO in 2019. This suggests, according to the WHO, that anemia in women of reproductive age in the DRC is a moderate public health problem. In 2019, WHO reported a prevalence of 42.4% for the DRC, which was comparable to some neighboring countries including Burundi with 38.5%, Rwanda with 17.2%, South Sudan with 35.6%, the Central African Republic with 46.8%, Tanzania with 38.9%, Uganda with 32.8%, Congo-Brazzaville with 48.8%, Angola with 44.5% and Zambia with 31.5% [22].

In our study, anemia in women of reproductive age evolves in an approximate inverse "U" shape with respect to age. Women aged 20–29 years and those aged 40 years and over have a higher risk of developing anemia than their counterparts under 20 years. These results corroborate those of Teshale A.B., et al. [4], Sunuwar D.R., et al. [32] and Messina JP et al. [33]. On the other hand, Kibret K.T. et al. found that women aged 40–49 years are less likely to be anemic than those aged 15–19 years [34].

Wouters H. et al. found that anemia is associated with decreased survival and health-related quality of life (HRQoL), particularly that related to physical health, in people over the age of 60 [35]. As a result, people with anemia are more likely to be frail, fatigued, cognitively impaired and have higher overall mortality than their non-anemic counterparts [36]. For their part, Steinmeyer Z. et al. found that low hemoglobin levels put elderly people at risk for poor oxygen delivery, exhaustion, fatigue and loss of muscle strength [37].

Pregnancy status determines a woman's risk of developing anemia during her reproductive years. Pregnant women have a lower risk of developing anemia than their non-pregnant counterparts. These results corroborate those of Teshale A.B., et al. [4], Gautam S., et al. [5], Sosa-Moreno A., et al. [33], Sunuwar D.R., et al. [32] and Messina JP et al. [38].

Maternal anemia is an important prenatal problem and should be investigated in pregnant women and candidates for pregnancy [39]. In 14 of 24 countries, including DRC, 40% or more of non-pregnant women were anemic [40]. The WHO estimates that 56% of all pregnant women in developing countries are anemic. In South Asia, the prevalence of anemia in pregnancy is about 75%, while in North America and Europe it is about 17%. In addition, 5% of pregnant women suffer from severe anemia in the most affected regions of the world [41]. Tirore L.L., et al. [42] believe that the higher risk of anemia in pregnant women may be due to the increased risk of infections and obstetric complications during pregnancy, which lead to blood loss and undernutrition.

The risk of anemia in women increases with increasing body mass index. Overweight and obese women have a higher risk of developing anemia than their normal weight counterparts, respectively. This is contrary to the results of the Quin Y., et al. [43] study which found that both overweight/obesity and central obesity are inversely associated with anemia.

Serum iron concentrations are lower with higher body mass index, particularly in women. The iron deficiency (DI) may result from the increased demand for iron in obese individuals due to their larger blood volume and consumption of energy-rich, nutrient-poor foods [9, 44].

Women's risk of anemia increases with increasing education level. Women with high school/university education have a lower risk of developing anemia than their counterparts without education. This contradicts the findings of Owais A., et al. [2] and Teshale A.B., et al. [4] who found that education level decreases with increasing risk of anemia.

Kumar P., et al. [45] believe that if both mother and father are educated, the probability of anemia in a family is low. But, if either the mother or the father is educated, this probability becomes low. UNESCO states that education alone is a health intervention. Educated people are better informed about specific diseases and act to prevent them at the first sign of illness. They use health services more effectively, feel more capable of achieving goals, and are more confident in their ability to make necessary lifestyle changes [46].

Education enables individuals, especially women, to live and aspire to healthy, meaningful, creative, and resilient lives. It strengthens their voice in community, national and global affairs. It opens up new work opportunities and sources of social mobility for them [47].

Ongoing breastfeeding determines the risk of anemia in women of reproductive age. Breastfeeding women have a higher risk of developing anemia compared to their non-breastfeeding counterparts. The results of this study corroborate those of Ali SA et al. [48], Gautam S [5], Min H, Kim H, Jeong H–S Sunuwar D.R. et al. [49], Nankinga O., and Aguta D. [50], and Habyarimana F., Zewotir T. and Ramroop S [51].

Anemia in the breastfeeding woman is due to inadequate vitamin intake. The "priority" nutrients for breastfeeding women are thiamine, riboflavin, vitamins B-6 and B-12, vitamin A and iodine [52, 53]. Kaliwile C., et al. [54] thought that vitamin intakes of rural Zambian women were inadequate. Calcium intake was higher in lactating women than in non-lactating women.

The use of contraceptive methods determines a woman's risk of developing anemia during her reproductive years. Women who use contraceptive methods to space births have a lower risk of developing anemia than their counterparts who do not use them. The results of this study corroborate those of Owais A., et al. [2], Teshale A.B., et al. [4], Gautam S., et al. [5], Sosa-Moreno A., et al. [33], Sunuwar D.R., et al. [32] and Messina JP et al. [38].

Hormonal contraceptive use is associated with a lower risk of anemia [2]. Teshale A.B., et al. [4] state that the use of modern contraceptive methods was associated with a 29% lower risk of anemia than women who did not use modern contraceptive methods. Being currently pregnant was associated with an 11% higher prevalence of anemia than non-pregnant women.

The use of insecticide-treated nets determines the risk of anemia in women of reproductive age. Women who sleep under insecticide-treated nets are more likely to develop anemia than their counterparts who do not use them. This is more relevant to the prevention of malaria in pregnant women [55].

ITNs have advantages for primigravida when used alone [56]. The main benefit of ITNs in women protected by IPTp-SP occurs after birth, through the protection of infants from malaria, as they usually share the sleeping space with the mother during the first months or years [57]. Esienumoh E., Mboho M., and Ndiok A. [58], state that, properly used, ITNs are an essential component in the prevention of malaria and its complications during pregnancy. Apart from its discomfort due to heat, odor, and difficulty in hanging, a mosquito net offers protection against mosquitoes and other insects, and thus against diseases such as malaria. With about 40% efficiency, the net protects the people sleeping under it and simultaneously kills the mosquitoes that come in contact with the net [40].

The consistent reduction observed in miscarriage and stillbirth rates suggests that the attributable effect of malaria on fetal loss may be underestimated in Africa, where malaria is endemic and most women remain asymptomatic when infected with P. falciparum. Despite the reduction in malaria infections, no overall effect on mean hemoglobin has been demonstrated, and data on maternal anemia are inconsistent [57]. For Inungu J.N., et al., although mass distribution of ITNs contributed to a high level of knowledge about malaria and the rapid achievement of high coverage rates, ITN use among pregnant women and children under five remained low [59]. Gamble C., et al. [57] estimate that women with low gravidity for IBD gave birth to fewer low birth weight babies and were less likely to experience fetal loss, i.e. miscarriage or stillbirth. Therefore, factors associated with ITN use need to be considered when designing evidence-based behavior change interventions to improve ITN use [58].

Household wealth affects women's anemia. Women from middle-income households are more likely to develop anemia than their counterparts from poor households. The results of this research contradict those of Teshale A.B., et al. [4], Kinyoki D., et al. [60] and Habyarimana F., Zewotir T., and Ramroop S. [51], who found that women in the lower category of the household wealth index are more anemic than those in the middle and upper categories of the wealth index. On the other hand, Kumar P., et al. [45] estimate that families from wealthy households were less likely to suffer from anemia than families from poor households.

An individual in a poor household cannot afford to pay for goods that would improve his or her health—better fuel, more nutritious food, and visits to the doctor—and that would therefore improve his or her ability to work. Thus, a vicious cycle where, because household members are both financially disadvantaged and in poor health, the household remains both in poverty and in poor health. In the economics literature, this phenomenon is referred to as the "poverty trap." [61].

Drinking water sources do not significantly affect women's anemia status. Women who get their drinking water from improved sources have a higher risk of developing anemia compared to their counterparts who get it from unimproved sources. This contrasts with the results of Gautam S., et al. [5] and Kothari M.T., et al. [62] who find that a strong relationship between sanitation facilities and anemia is also observed in most countries. Lack of access to safe water at the site increases the risk of anemia prevalence in children and women.

Sanitation facilities have a negative impact on women's anemia status. Women using improved latrines are less likely to develop anemia than their counterparts using unimproved latrines. The results of this study corroborate those of Teshale A.B., et al. [4], who found that women from households with unimproved toilets and unimproved water sources had a higher prevalence of anemia than their counterparts.

Universal, affordable, and sustainable access to WASH practices (safe water, adequate sanitation, and hygiene education) can reduce disease and death and improve health outcomes at the population level [22].

## Conclusion

The innovation of our study is the inclusion of the multilevel and spatial nature of multifactorial risk factors of anemia among women of reproductive health in DRC using robust spatial Bayesian ordinal logistic regression statistical model that is able to disentangle individual level factors such as age, pregnancy status, body mass index, education level, current breastfeeding, current marital status, use of contraceptives and insecticide-treated mosquito nets, source of drinking water, use of toilets or latrines from community and environmental factors such province of residence as risk factors for anemia in women of reproductive age in DRC.

## Data Availability

The datasets generated and/or analyzed during the current study are available in the database of the Second Demographic and Health Survey of the Democratic Republic of Congo of 2013–2014 (DHS-DRC II) is officially downloadable on the net from "The DHS Program-Congo Democratic Republic: Standard DHS, 2013–14". "The datasets used and/or analysed during the current study available from the corresponding author on reasonable request."
